# The DNMT1/PCNA/UHRF1 disruption induces tumorigenesis characterized by similar genetic and epigenetic signatures

**DOI:** 10.1038/srep04230

**Published:** 2014-03-18

**Authors:** Romain Pacaud, Emeline Brocard, Lisenn Lalier, Eric Hervouet, François M. Vallette, Pierre-François Cartron

**Affiliations:** 1 Centre de Recherche en Cancérologie Nantes-Angers, INSERM, U892, Equipe Apoptose et progression tumorale, Equipe labellisée Ligue Nationale Contre le Cancer. 8 quai moncousu, BP7021, 44007 Nantes, France; 2 Université de Nantes, Faculté de Médecine, Département de Recherche en Cancérologie, IFR26, F-4400, Nantes, France; 3 Université de Franche-Comté, Equipe EA3922, 16 Route de Gray, 25035 Besançon Cedex, France; 4 LaBCT, Institut de Cancérologie de l'Ouest, Boulevard J Monod, 44805 Nantes, Saint Herblain Cedex, France; 5 Membre du réseau Epigénétique du Cancéropole Grand-Ouest

## Abstract

Several genetic and epigenetic signatures characterize cancer cells. However, the relationships (causal or consequence link, existence due to a same origin) between these 2 types of signatures were not fully elucidated. In the present work, we reported that the disruption of the DNMT1/PCNA/UHRF1 complex acts as an oncogenic event of the tumor transformation of brain (astrocytes), breast, lung and mesothelial cells. We also show that these tumor transformation processes were associated with the acquisition of cancer hallmark and common genetic and epigenetic signatures. Thus, our data revealed that the global DNA hypomethylation induced by the DNMT1/PCNA/UHRF1 disruption is an oncogenic event of human tumorigenesis, an inducer of epigenetic and genetic signatures frequently observed in several human cancers, and is an initiator of oncogenic events.

DNA methylation is an epigenetic feature influencing cellular development and function, and aberrations of DNA methylation are a candidate mechanism for the development of cancer^1^. Indeed, we and other have described that the DNMT1/PCNA/UHRF1 complex plays a crucial role in maintaining DNA methylation in mammalian cells and that its disruption acts as an oncogenic event of gliomagenesis^2^,^3^,^4^,^5^.

Based on this last point, we firstly asked whether the disruption of the DNMT1/PCNA/UHRF1 complex could also promote the tumor transformation of a panel of non-tumorigenic cell lines such as astrocytes (Astro#40), mammary epithelial cells (MCF10A), lung fibroblast cells (Wi38) and mesothelial cells (Met5A).

## Results

In a previous work, we demonstrated that the disruption of the DNMT1/PCNA/UHRF1 complex promotes the global DNA hypomethylation phenotype and the gliomagenesis^2^,^4^.

**Figure 1:**
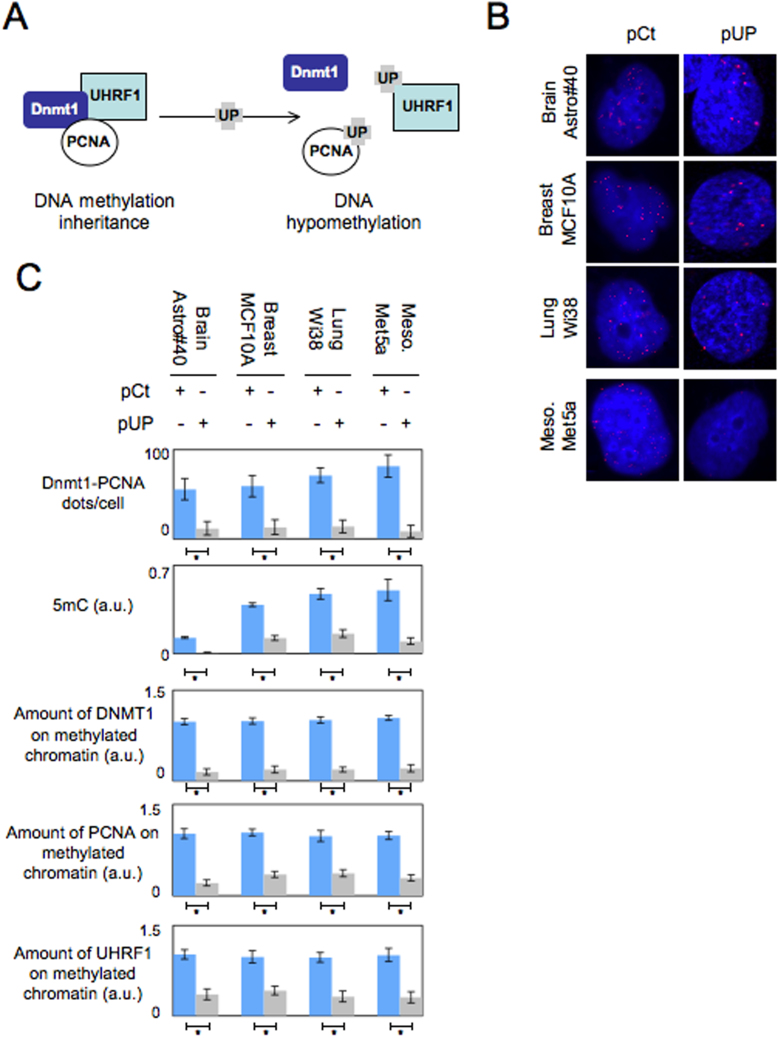
The DNMT1/PCNA/UHRF1 disruption in brain, lung, mammary and mesothelioma cells induces global DNA hypomethylation. (A) Schematic representation of the effect of the UP chimera protein on the DNMT1/PCNA/UHRF1 complex. (B) Proximity ligation *in situ* assay (P-LISA) monitoring the number of DNMT1/PCNA dots in Astro#40 (Clonexpress, Gaithersburg, US), MCF10A (ATCC, CRL-10317), Wi38 (ATCC, CRL-75) and Met5A (ATCC, CRL-9444) after transfection by the pUP or pCt (Control) plasmid. Pictures are obtained by using ApoTome microscopic system. One red dot represents one DNMT1/PCNA interaction. (C) Graphs illustrate the number of DNMT1/PCNA dots according to the P-LISA analyses, the 5-methylcytosine number via the use of the Methylamp Global DNA Methylation Quantification kit (Epigentek-Euromedex, France), and the expression level of DNMT1 and PCNA (ELISA methods). The amount of DNMT1, UHRF1 and PCNA recruited on methylated chromatin was determined by ELISA realized from methylated chromatin issue to MeDIP (Active Motif, France). *: p < 0.05.

To perform the disruption of the DNMT1/PCNA/UHRF1 complex, we used the UP chimera protein encoded by the pUP plasmid since this protein is composed of the amino-acid regions in DNMT1 that interact with PCNA (163–174) and UHRF1 (596–614) (Figures 1A and S1). The specific of action of the UP chimera protein was validated by the fact that the mutated forms did not disrupt the DNMT1/PCNA interaction in acellular system (Figures S1 and S2). The nuclear localization of the UP and UP^mutated^ proteins was validated by a radiolabelled UP protein obtained by *in vitro* tanslation method that was detected in nuclear extract (Figure S3).

To determine whether the disruption of the DNMT1/PCNA/UHRF1 complex in other cells could promote the global DNA hypomethylation phenotype, we disrupted this complex in astrocytes (Astro#40), mammary epithelial cells (MCF10A), lung fibroblast cells (Wi38) and mesothelial cells (Met5A) by disrupting the DNMT1/PCNA and DNMT1/UHRF1 interactions. After successful transfections (Figure S1), DNMT1/PCNA Proximity Ligation *In Situ* Assay (P-LISA) revealed a strong reduction in the DNMT1/PCNA dots in all cells transfected with the pUP plasmid (Figures 1B and 1C). Co-immunoprecipitation experiments also confirmed that DNMT1/PCNA and DNMT1/UHRF1 interactions were reduced in cells transfected with pUP plasmid (Figure S4).

By performing ELISA to monitor the level of 5-methylcytosine (5 mC), we observed that the 5 mC level decreased in cells transfected with the pUP plasmid (Figure 1C). Thus, as expected, we noted that the disruption of the DNMT1/PCNA interactions by the UP chimera protein in Astro#40, MCF10A, Wi38 and Met5A cells promoted the acquisition, by these cells, of a global DNA hypomethylation phenotype. In addition, ELISA monitoring the expression of the DNMT1, PCNA and UHRF1 proteins indicated that cells transfected with the pUP plasmid or pCT plasmid (control) expressed similar levels of DNMT1, UHRF1 and PCNA (Figure S5). Thus, we conclude that the global DNA hypomethylation induced by the disruption of the DNMT1/PCNA interactions by the UP chimera protein is not caused by a loss of expression of the DNMT1, UHRF1 and PCNA proteins.

We then analyzed whether the disruption of the DNMT1/PCNA interactions by the UP chimera protein could promote the decrease of the DNMT1, UHRF1 and PCNA recruitment on chromatin. For this purpose, we quantified by ELISA the amount of DNMT1, PCNA and UHRF1 associated with methylated chromatin. Methylated chromatin was isolated using MeDIP methods, and the amount of DNMT1, UHRF1 and PCNA purified from methylated chromatin was determined by ELISA. Figure 1C shows that DNMT1, PCNA and UHRF1 had a reduced recrutment to methylated chromatin in cells transfected with the pUP plasmid than in cells transfected with pCT. Thus, we conclude that the disruption of the DNMT1/PCNA interaction by the UP chimera protein promotes a decrease in the recruitment of DNMT1, PCNA and UHRF1 to methylated chromatin.

**Figure 2:**
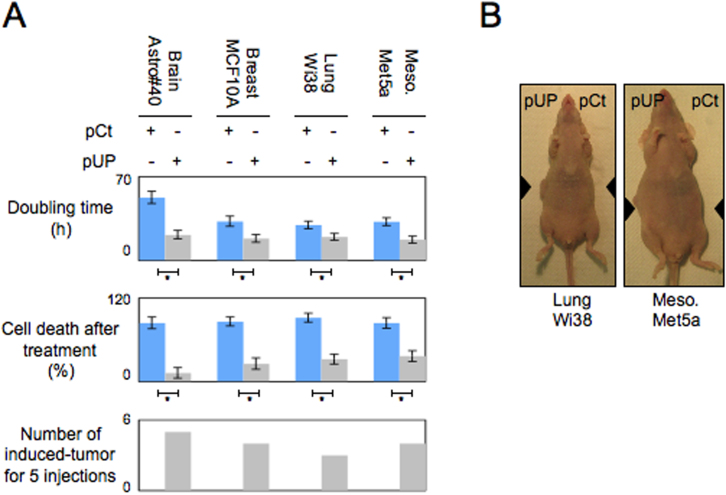
The DNMT1/PCNA/UHRF1 disruption in brain, lung, mammary and mesothelioma cells induces the acquisition of cancer hallmarks and promotes the tumorigenesis. (A) Graphs illustrate the doubling time of the considered cells, the cell death percentage after irradiation (trypan blue method, irradiation: 5 Gy) and the number of tumor seen for 5 injections of indicated cells. Doubling time (i.e. the period of time required for a quantity to double in size) was calculated by using the Doubling Time Online Calculator website (Roth V. 2006, http://www.doubling-time.com/compute.php) and counting the proliferation of 10^3^ cells during 120 hours. *: p < 0.05 (B) Picture illustrating the tumorigenicity of the Wi38-UP and Met5A-UP cells.

We next searched whether the acquisition of the global DNA hypomethytion phenotype by the Astro#40, MCF10A, Wi38 and Met5A cells transfected with the pUP plasmid was associated with the acquisition of cancer phenotypes such as resistance to cell death, high proliferation rate or tumorigenic capacity. By comparing the doubling time and the cell death percentage in response to irradiation treatment of our cells (Figure S6), we observed that all cells transfected with the pUP plasmid were more proliferative and more resistant to irradiation-induced cell death than the corresponding cells transfected with the pCt plasmid (control) (Figure 2A).

In our tumorigenic assay, we used the term “tumor formation” to define the detection of tumors whose the volume was equal or superior to 200 mm^2^. According to the guidelines of Institutional Animal Care and the French National Committee of Ethics, we performed our tumorigenic assay 13 weeks after the injection of cells.

As illustrated by the figures 2A and 2B, we noted that 5/5 injections Astro#40-UP cells, 4/5 injections of MCF10A-UP cells 4/5 injections Met5A-UP cells and 3/5 injections Wi38-UP cells promoted the formation of tumors, while injections with the corresponding control cells not promoted tumor formation. By analyzing the kinetics of tumor formation, we noted that the majority of tumor formation occurred three weeks after the injection of cells transfected with the pUP plasmid (Figure S7). Thus, all these data indicate that the induction of the global DNA hypomethylation via the disruption of the DNMT1/PCNA interactions is a process associated with the acquisition by astroctyes, mammalian epithelial cells, lung fibroblast and mesothelial cells of three phenotypes of cancer: high proliferation rate, resistance to cell death and tumorigenic capacity. In other terms, our data suggest that the global DNA hypomethylation induced by the disruption of DNMT1/PCNA interactions acts such as an oncogenic event of the brain, breast, mesothelial and lung cancers. Besides, the idea that the decrease of the DNMT1/PCNA interactions could be at the origin of the tumor transformation associated with the acquisition of the global DNA hypomethylation phenotype in breast cancer is also supported by the fact that several malignant breast cell lines harbor a lower level of DNA methylation and of DNMT1/PCNA interactions than the MCF10A non-tumorigenic breast cell line^6^.

Global DNA hypomethylation potentially affecting certain genes, we next searched to identify whether the global DNA hypomethylation induced by the pUP plasmid in Astro#40, MCF10A, Wi38 and Met5A cells could promote the hypomethylation of genes clusters. For this purpose, we used the TransSignal™ Promoter Methylation Array (Ozyme-Panomics, France), which analyzes the methylation status of 82 different promoter regions (−900/+200).

**Figure 3:**
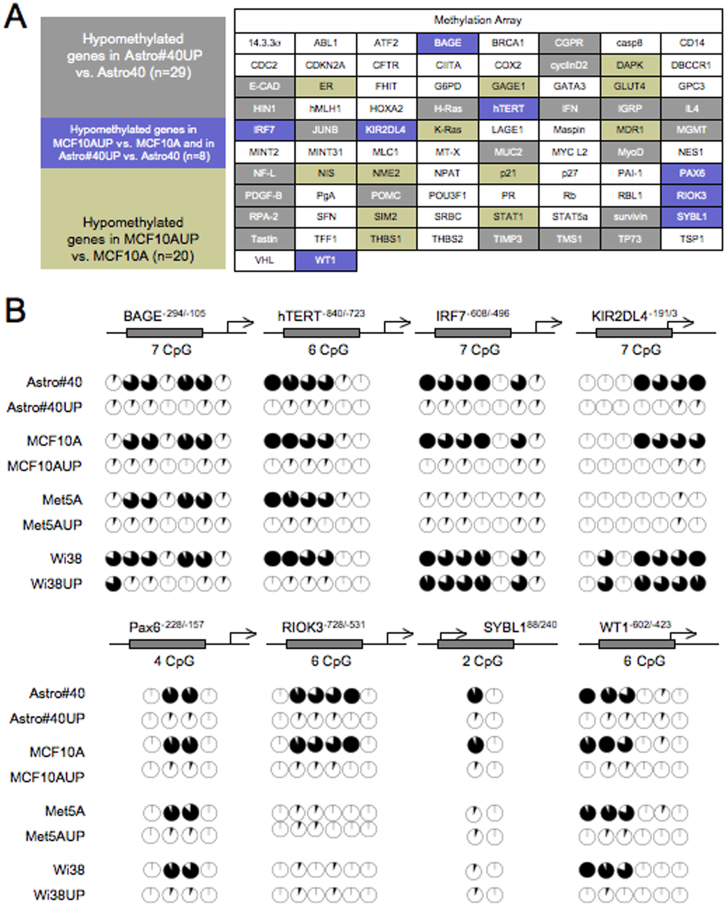
The global DNA hypomethylation induced by DNMT1/PCNA/UHRF1 disruption in brain, lung, mammary and mesothelioma cells is associated with common epigenetics aberrations. (A) Epigenetic signatures commonly seen in Astro#40-UP and MCF10A-UP cells were initially obtained by comparing Promoter Methylation Array (Ozyme, France) data obtained for these cells (according to our previous results^2^). (B) Hypomethylation signatures seen in Astro#40UP and MCF10A-UP were searched in Wi38/Wi38-UP cells and Met5A/Met5A-UP cells by using using bisulfite sequencing method. Each circle graph illustrates the percentage of methylation of a CG of interest. The percentage of methylation was calculated by considering 15 sequencing. In circle diagram, black color represents the methylation percentage, and white color represents the unmethylation percentage.

By comparing the results of TransSignal™ Promoter Methylation Array obtained for the Astro#40/Astro#40-UP and MCF10A/MCF10A-UP cells, we established a list of 8/82 genes commonly hypomethylated in the Astro#40-UP and MCF10A-UP cells (Figure 3A). Next, Bisulfite sequencing analyses confirmed that the *BAGE, hTERT, IRF7, KIR2DL4, Pax6, RIOK3, SYBL1* and *WT1* genes were hypomethylated in the Astro#40-UP and MCF10A-UP cells, and that the *BAGE, hTERT, Pax6*, and *WT1* genes were commonly hypomethylated in Astro#40-UP, MCF10A-UP, Wi38-UP and Met5A-UP cells (Figure 3B). Thus, these data indicate that the disruption of the DNMT1/PCNA/UHRF1 complex via the UP chimera protein promoted the common hypomethylation, in the Astro#40, MCF10A, Wi38 and Met5A cells, of less than 5% of the genes considered in our study. However, this percentage needs to be relativized/discussed by the fact that the Astro#40, MCF10A, Wi38 and Met5A cells harbor different methylome and that the DNMT1/PCNA/UHRF1 complex does not promote a process of specific DNA methylation inheritance in terms of oligonucleotidique sequence^7^.

**Figure 4:**
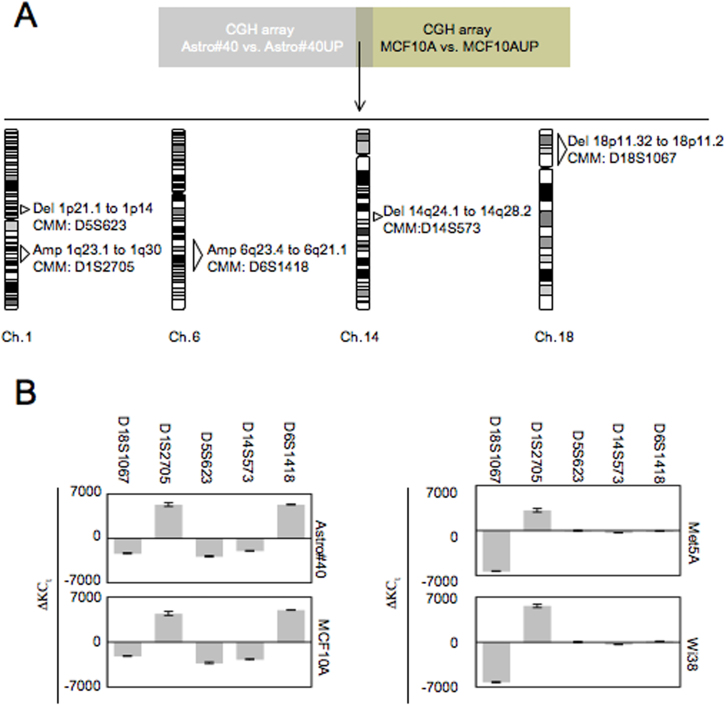
The global DNA hypomethylation induced by DNMT1/PCNA/UHRF1 disruption in brain, lung, mammary and mesothelioma cells is associated with common genetics aberrations. (A) Genetic signatures commonly seen in Astro#40-UP and MCF10A-UP cells were initially obtained by comparing CGH array data obtained for these cells (according to our previous results^2^). (B) Common genetic signatures were searched in Wi38/Wi38-UP cells and Met5A/Met5A-UP cells by using adequate chromosome map marker (such as referred in figure). Fold copy number change (ΔKC_t_) was calculated according to previous reports^9^. ΔKC_t_ values of 0 indicate an equal ratio of the target and reference, which corresponds to no chromosomal amplification or deletion, ΔKC_t_ < 0 values indicate the presence of chromosomal deletion at the corresponding chromosome marker, ΔKC_t_ > 0 values indicate the presence of chromosomal amplification at the corresponding chromosome marker.

Global DNA hypomethylation inducing chromosomal aberrations, we next investigated whether the Astro#40-UP, MCF10A-UP, Wi38-UP and Met5A-UP cells could be characterized by similar chromosomal aberrations^2^,^8^. To investigate this point and according to our previous reports, we firstly established a list of chromosomal aberrations commonly occurring in the Astro#40-UP and MCF10A-UP cells by comparing the CGH array data obtained for these cells (Figure 4A)^2^,^4^. Thus, we observed that the presence of pUP plasmid in Astro#40 and MCF10A cells promoted five similar chromosomal aberrations: three deletions (Del 1p21.1 to 1p14, Del 14q24.1 to 14q28.2 and Del 18p11.32 to 18p11.2) and two amplifications (1q23.1 to 1q30 and 6q23.4 to 6q21.1). Then, these chromosomal aberrations have been searched in the Wi38/Wi38-UP and Met5A/Met5A-UP cells by using adequate chromosome map marker and qPCR method^9^. The use of the D18S1067 and D1S2705 chromosome map markers revealed the presence of a chromosomal deletion affecting the 18p11.2 region (del-18p11.2) and a chromosomal amplification affecting the 1q23.3 region (amp-1q23.3) in the Astro#40-UP, MCF10A-UP, Wi38-UP and Met5A-UP cells (Figure 4B). Thus, these data associated the global DNA hypomethylation induced by the disruption of the DNMT1/PCNA interactions with the presence of the del-18p11.2 and the amp-1q23.3 in the Astro#40-UP, MCF10A-UP, Wi38-UP and Met5A-UP cells.

To summarize, it appears that our data associate the disruption of the DNMT1/PCNA interaction with the presence of a global DNA hypomethylation phenotype, the acquisition of several cancer phenotypes, the hypomethylation of certain genes and with the presence of chromosomal aberrations.

**Figure 5:**
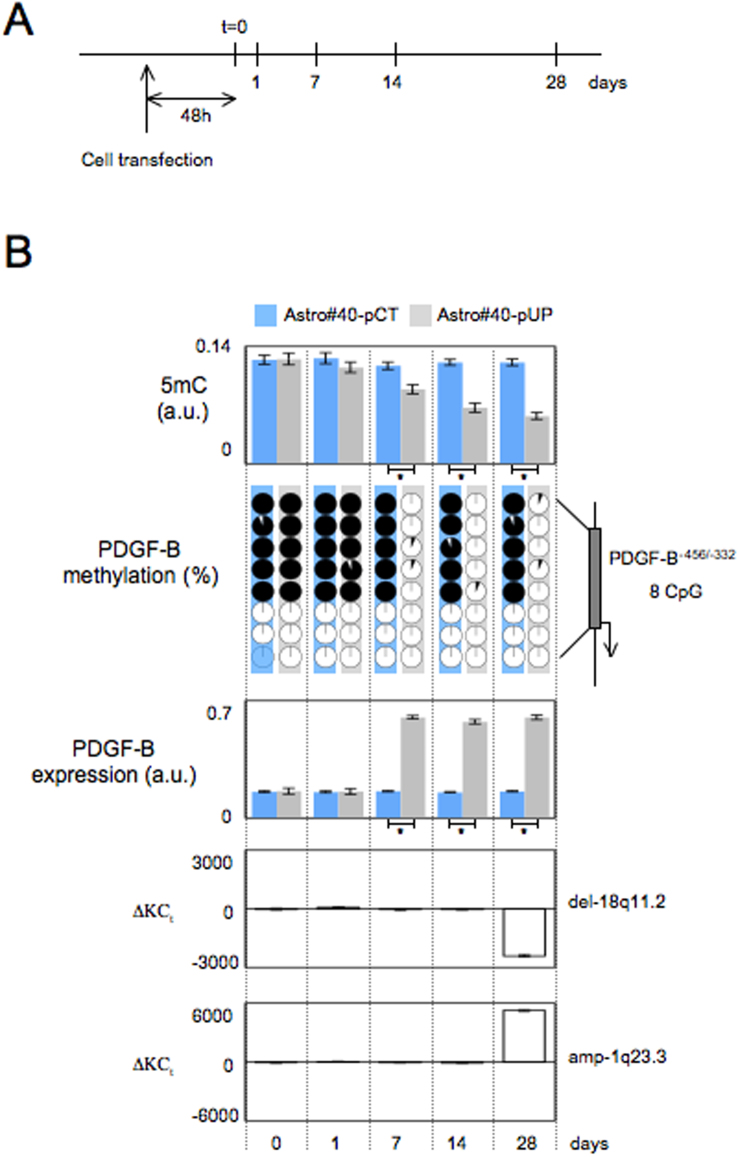
Kinetics of epigenetic and genetics modifications occurring in Astro#40-UP cells. (A) Schematic representation of experiments. (B) Monitoring of epigenetic and genetics modifications in Astro#40-UP and Astro#40-Ct cells. The level of 5methylcytosine (5 mC) was evaluated by ELISA method (Methylamp Global DNA Methylation Quantification kit, Epigentek-Euromedex, France). The methylation status of *PDGF-B* gene was estimated by using using bisulfite sequencing method. Each circle graph illustrates the percentage of methylation of a CG of interest. The percentage of methylation was calculated by considering 15 sequencing. In circle diagram, black color represents the methylation percentage, and white color represents the unmethylation percentage. The PDGF-B expression level was measured by using the PDGF BB Human ELISA Kit (Abcam, France). The genetic modifications (del-18p11.2 and amp-1q23.3) were analyzed by qPCR method. Fold copy number change (ΔKC_t_) was calculated according to previous description. *: p < 0.05.

By focusing on the Astro#40 and Astro#40-UP model, we then analyzed the kinetics of the appearance of certain epigenetic and genetic events associated with the disruption of the DNMT1/PCNA interaction, which we monitored by the global 5 mC level, the methylation status, the expression of the *PDGF-B* gene (since PDGF-B is an oncogene of gliomagenesis^10^), and the del-18p11.2 and amp-1q23.3 (Figure 5A). Our analyses indicated that the global 5 mC level and the methylation level of *PDGF-B* decreased at day#7, while the del-18p11.2 and amp-1q23.3 were detected at day#28 (Figure 5B). ELISA analyses indicated an elevation of PDGF-B expression level at day#7 i.e. when the methylation level of the *PDGF-B* gene decreased (Figure 5B). Supported by these data, we noted that epigenetic modifications occurred before genetic aberrations.

**Figure 6:**
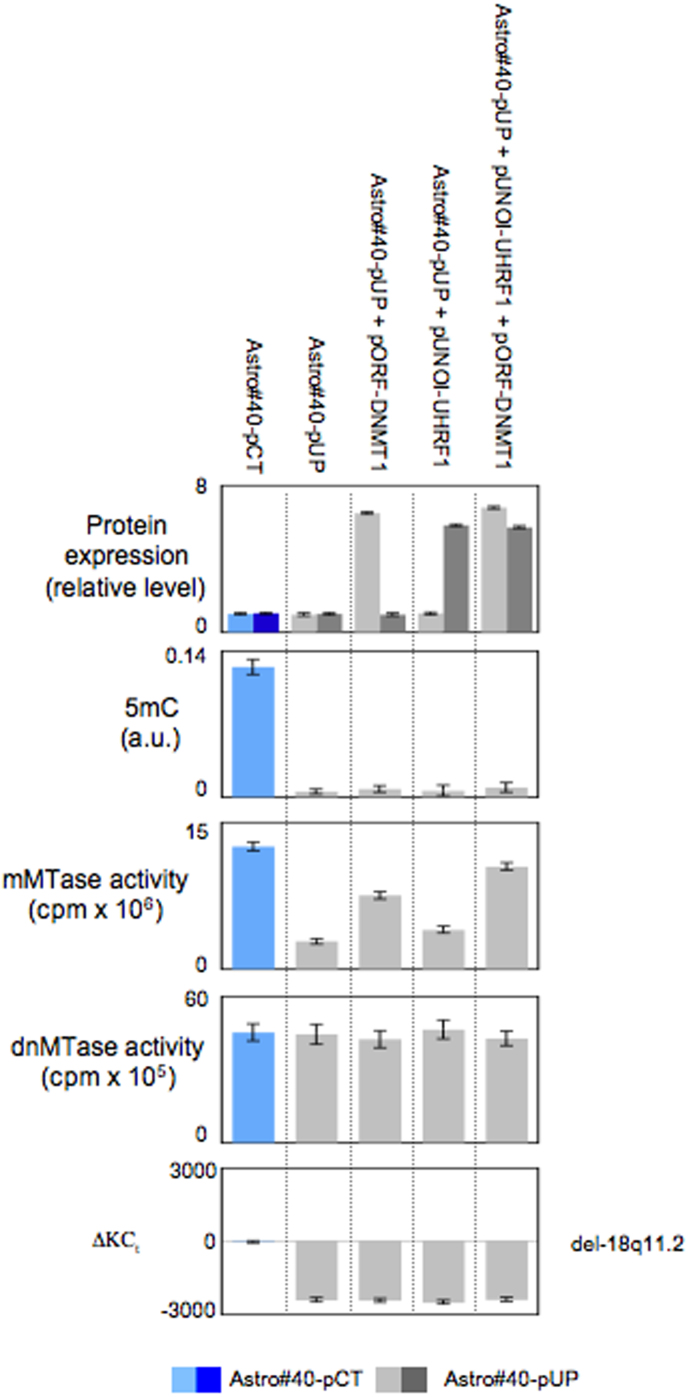
Impact of the DNMT1 and/or UHRF1 overexpression on oncogenic effect induced by the expression of the pUP plasmid. Cell tranfections were realized by using 2.10^5^ cells, 5 μg of plasmid (In vivogen, France) and Lipofectamine™ 2000 reagents (Life Technology, France). Selection was realized by adding 500 mg/ml of selective antibiotic in complete medium of cell culture for 3 weeks. Next, 1 week was used to amplify the cells and tests were realized. Level of 5methylcytosine (5 mC) was measured via the use of the Methylamp Global DNA Methylation Quantification kit (Epigentek-Euromedex, France). The maintenance (mMTase) and *de novo* methylatransferase (deMTase) activities were measured by using DMB assay as previously reported^2^,^31^. Del-18q11.2 was monitored as previously described.

Finally, we investigated whether the overexpression of DNMT1 and/or UHRF1 could abolish certain oncogenic signatures induced by expression of the pUP plasmid. ELISA results indicated that the stable transfection of Astro#40-pUP cells with the pORF-DNMT1 and/or p-UNOI-UHRF1 plasmids promoted the overexpression of the DNMT1 and UHRF1 proteins (near to 7- and 6-fold, respectively) (Figure 6). None significant variation in 5 mC was observed in Astro#40-UP cells transfected with the pORF-DNMT1 and/or p-UNOI-UHRF1 plasmids (Figure 6). Interestingly, we noted that this observation was consistent with the fact that cells overexpressing DNMT1 harbored an increase in maintenance methyltransferase (mMTase) activity but not an increase in *de novo* methyltransferase (deMTase) activity i.e. the activity implicated in the process of gain of methylation (Figure 6).In parallel, we noted that del-18q11.2 persisted in Astro#40-UP cells transfected with the pORF-DNMT1 and/or p-UNOI-UHRF1 plasmids (Figure 6).

Thus, these experiments indicated that the DNMT1 and/or UHRF1 overexpression have the ability to increase the mMTase activity of Astro#40-UP cells, but this increase is not sufficient to suppress oncogenic events induced by the expression of pUP plasmid such as the global DNA hypomethylation and a genomic aberrations (del-18q11.2).

## Discussion

Taken together our work provides a strong proof of concept to the fact that the global DNA hypomethylation induced by the disruption of the DNMT1/PCNA interactions can act as an oncogenic event of mammalian tumorogenesis. Moreover, DNMT1/PCNA disruption-induced tumors harbor a set of similar genetic and epigenetic (gene-specific hypomethylation) signatures. In addition, our data indicated that the presence of these commune genetic and epigenetic signatures occurred independently of the concerned cell types since we used, in our experiments, non-tumorigenic astrocytes (Astro#40), a mesothelial cell line obtained from a non-cancerous individual (Met5A), lung fibroblasts (Wi38) and a non-tumorigenic breast cell line (MCF10A). The use of these cells and other human non-tumoral cells also confirmed the fact that the “UP treatment” induced a global DNA hypomethylation phenotype in any cell type (Figure S8).

Other processes can also promote the global DNA hypomethylation: deletion of DNMT1, UHRF1, or treatment with inhibitors of DNA methyltransferases (such as 5-aza-2-deoxycytidine - 5aza). Thus, we can suppose that these processes could also induce tumorigenesis. To investigate this point, tumorigenic assays were performed with Astro#40 cells expressing the pUP plasmid, treated with 5aza, and harboring a sh-RNA-induced down-expression of DNMT1 or UHRF1 (Figure S9). Thus, we noted that the 5-aza treatment promoted tumorigenesis in 60% of cases (3/5), while the partial depletion of DNMT1 or UHRF1 did not promoted the tumorigenesis (Figure S9). Interestingly, we noted that tumorigenesis appeared when global DNA hypomethylation was higher (cases of the UP-induced DNMT1/PCNA/UHRF1 disruption and 5-aza treatment), and not when the global DNA hypomethylation was lower (cases of the sh-RNA-induced depletion of DNMT1 or UHRF1) (Figure S9).

Concerning the use of 5-aza, we are conscious that the tumorogenesis processes associated with the use of this drug are obtained after a long exposure. However, 4 weeks is not a long period on the scale of the majority of the chemotherapeutic treatments that extend over several months. Regardless, our data provide “a warning” for the use of DNA demethylating agent as an anti-cancer therapy, and provide proof reinforcing the necessity to use specific DNMT inhibitors or unspecific DNMT1 inhibitors with an adequate and optimized dose-schedule such as already described by several publications^11^,^12^,^13^.

Among the genetic aberrations commonly seen in Astro#40-UP, MCF10A-UP, Wi38-UP and Met5A-UP cells, we noted that literature described the del-18p11 as being a chromosomal deletion frequently observed in glioma, mesothelioma, lung and breast cancer and that this deletion can predicts an adverse clinical outcome in patients with high-risk breast cancer^14^,^15^,^16^,^17^. According to the literature, we also noted that the del-18p11 could affect the loss of expression of Gata-6, a tumor suppressor gene of gliomagenesis; and that the amp-1q23.3 could be at the origin of the Intelectin-1 high expression seen in mesothelioma^18^,^19^.

Concerning the genes specifically hypomethylated during the tumorigenesis induced by the disruption of the DNMT1/PCNA interaction in Astro#40, MCF10A, Wi38 and Met5A cells, literature mentions the fact that the hypomethylation of the *BAGE* gene can be used as a biomarker for cancer detection, that Il6-induced tumorigenesis can promoted the hypomethylation of the *Pax-6* gene^20^,^21^.

The presence of hypomethylated genes asks the question of the expression level of these genes (especially if these genes are tumor suppressor genes or oncogenes). By focusing on MCF10A and MCF10A-UP, we observed that the hypomethylation of the *hTERT, K-ras*, and *MDR1* genes was a process associated with the overexpression of proteins encoded by these genes, while the hypomethylation of the *p21* and *WT1* genes had no effect on the expression level of proteins encoded by these genes (Figure S10). Thus, we noted that the hypomethylation of a gene of interest is not obligatory associated with its overexpression. In our example, we also observed that, among the hypomethylated and overexpressed genes; *K-ras*, an oncogene (according to the CancerQuest website (Emory University, http://www.cancerquest.org/oncogene-table.html), hTERT (the catalytic subunit of telomerase), a protein favoring an immortal phenotype by blocking programmed cell death (apoptosis) independently of its protective function at the telomere ends^22^, and MDR1 a energy-dependent efflux pump responsible for decreased drug accumulation in multidrug-resistant cells^23^. In other terms, we noted that all hypomethylated and overexpressed genes highlighted in our work could play a crucial role in the tumorigenesis initiated by the disruption of the DNMT1/PCNA interaction.

By focusing on the Astro#40/Astro#40-UP model, our data indicated that epigenetic modifications (decrease of 5 mC level and hypomethylation of *PDGF-B* gene) occurred before genetics modifications (del-18p11.2 and amp-1q23.3). In addition, we also noted that the *PDGF-B* hypomethylation is associated with the PDGF-B overexpression. Thus, these results suggest the existence of a chronology of events implicated in the DNMT1/PCNA disruption-induced tumorigenesis: loss of DNMT1/PCNA interaction, global and gene-specific hypomethylation (*PDGF-B* and *K-ras*), oncogene expression (PDGF-B and K-ras) and genetic aberrations (del-18p11.2 and amp-1q23.3). Thus, our data suggest that the DNMT1/PCNA/UHRF1 disruption is a process able to initiate oncogenic events such as: global DNA hypomethylation, (over)expression of oncogene and chromosomal abnormally.

Finally, by observing that the overexpression of DNMT1 and/or UHRF1 did not suppress the oncogenic effect of the pUP plasmid (i.e. of the disruption of the DNMT1/PCNA/UHRF1 interactions), our data reinforce the idea that the integrity of the DNMT1/PCNA/UHRF1 complex is crucial in the tumor transformation. The fact that the overexpression of DNMT1 and/or UHRF1 did not suppress the oncogenic effect of the pUP plasmid is consistent with the fact that 1) the oncogenic effect of pUP plasmid includes genetic aberrations, and genetic aberrations are not reversible; and 2) to promote a remethylation replying to the DNA hypomethylation induced by the pUP plasmid, we need to stimulate the machinery of *de novo* methylation. Now, the DNMT1 and/or UHRF1 overexpression increases the maintenance methyltransferase activity and not the *de novo* methyltransferase activity (which must be increase to promote a remethylation).

To summarize, our data are ones of the first to report that the global DNA hypomethylation induced by the DNMT1/PCNA/UHRF1 disruption is an oncogenic event of several human tumorigenesis, an inducer of epigenetic and genetic signatures frequently observed in several human cancers, and is a process initiator of oncogenic events. Our data also reinforced the idea that DNMT1/PCNA/UHRF1 play a crucial role in the inheritance of DNA methylation, and that the disruption of this complex plays a crucial role in the tumor transformation process^2^,^3^,^5^. However, despite the crucial character of the integrity of the DNMT1/PCNA/UHRF1 complex, the integrity of other DNMT1 including complexes plays a major role in the inheritance of DNA methylation and in tumor development and progression. Indeed several publications refer to the fact that the integrity of DNMT1 is essential in the DNA methylation inheritance and not its interaction with replication machinery, and that the disruption of other interactions existing between DNMT1 and protein binding DNMT1 plays a role in tumor development and progression^24^,^25^,^26^. Nevertheless, to date, the disruption of the DNMT1/PCNA/UHRF1 complex is one of the first disruptions of an epigenetic player identified as having the ability to promote the tumor transformation of several cells types (astrocytes, mammary epithelial cells, lung fibroblast cells and mesothelioma cells).

## Methods

### Proximity Ligation In Situ Assay (P-LISA)

P-LISA is a technology permitting to visualize stable and transient interactions at endogenous protein levels directly *in situ*^27^. Briefly, two primary antibodies raised in different species recognize the target antigen or antigens of interest. Species-specific secondary antibodies, called PLA probes, each with a unique short DNA strand attached to it, bind to the primary antibodies. When the PLA probes are in close proximity (<40 nm), the DNA strands can interact through a subsequent addition of two other circle-forming DNA oligonucleotides. After ligating the two added oligonucleotides creating a circle DNA molecule, they are amplified via rolling circle amplification. After amplification, several-hundredfold replication of the DNA circle has occurred, and labeled complementary oligonucleotide probes highlight the product. The resulting high concentration of fluorescence in each single-molecule amplification product is easily visible as a distinct bright dot when viewed with a fluorescence microscope.

Cells were cultured for 24 h on a cover slip. Cells were then fixed with 4% paraformaldehyde in PBS pH 7.4 for 15 min at room temperature. Permeabilization was performed with PBS containing 0.5% Triton X-100 for 20 min at room temperature. Blocking, staining, hybridization, ligation, amplification and detection steps were realized according to manufacturer's instructions (Olink Bioscience). All incubations were performed in a humidity chamber. Amplification and detection steps were performed in dark room. Fluorescence was visualized using an Axiovert 200 M microscopy system (Zeiss, Le Pecq, France) with ApoTome module (X63 and numerial aperture 1.4). Preparations were mounted using ProLong® Gold antifade reagent with DAPI (Invitrogen, France). Pictures acquisition was realized in structured illumination microscope^28^. Finally, the images were analyzed by using the freeware “BlobFinder” available for download from www.cb.uu.se/~amin/BlobFinder to identify nuclei automatically obtaining the number of signals per nuclei.

### Methylation analyses

#### Measure of the 5-methylcytosine level

DNA was extracted using the QiaAmp DNA mini Kit (Qiagen, France). The quantification of 5-methylcytosine is performed by using the Methylamp Global DNA methylation Quantification kit (Euromedex-Epigenetiek, France).

#### Bisulfite methylation sequencing

Bisulfite methylation sequencing was used to confirm the methylation patterns of genes of interest^29^,^30^. Bisulfite conversion was performed with the MethylDetector bisulfite modification kit (Active Motif, France). PCR fragments were cloned using the TopoTA cloning kit (Life Technology, France). 15 single colonies carrying individual DNA molecules were then picked and the plasmid DNA sequenced. If the CpG dinucleotide was methylated in the original DNA, the sequencing read will show a CG at that location. If the CpG dinucleotide was unmethylated in the original DNA, the bisulfite conversion will have converted the cytosine to uracil and the sequencing read will show a TG at that location. By comparing the bisulfite converted sequence data against the unconverted DNA sequence, we quantified the number of methylation site for the region of interest to determine the percentage of methylation. This percentage was represented in circle graph.

### Tumorigenicity assay and mice treatment

Cultured cells were harvested by trypsinization, washed and resuspended in saline buffer. Cell suspensions were injected s.c. as 10^6^ cells in 0.2 ml volume in the flank of 7-/8-week-old nude NMRI-nu female mice (Janvier, France).

The experimental procedures using animals were in accordance with the guidelines of Institutional Animal Care and the French National Committee of Ethics. In addition, all experiments were conducted according to the Regulations for Animal Experimentation at the « Plate-forme Animalerie » of « Institut de Recherche en Santé de l'Université de Nantes (IRS-UN) » and approved by the French National Committee of Ethics (Aggrement number: B44278).

### Preparation of the UP-cells

The UP-cells were obtained after nucleofection by using the Nucleofector™ kit (Lonza biosystems, France). After 4 weeks of selection in presence of Neomycin, PCRs were performed to detect the integration of insert/constructs in genomic DNA (according to our previous publication^2^,^4^). Positive cells were expanded for 2 weeks and used for analyses.

### Promoter methylation array analysis

To analyze the methylation status of several genes, we used the TransSignal™ Promoter Methylation Array (Panomics/Ozyme France). According to the manufacturer's instructions, fours steps are involved: (1) Genomic DNA digested with a restriction enzyme (MseI) to isolate DNA with CpG islands. The digests are purified and adapted with linkers. (2) The adapted DNA is then incubated with the methylation binding protein, which forms a protein/DNA complex. These complexes are separated and methylated DNA is isolated. (3) The methylated DNA is labeled with biotin-dCTP via PCR and these probes are hybridized to the methylation array. (4) Detection by using streptavidin-HRP conjugate.

For each assay, we used 5 μg Genomic DNA extracted by using the NucleoSpin® Tissue kit (Macherey-Nagel, France).

### Genetic analyses

Array-CGH experiments were performed by PartnerChip (Evry, France) using the Constitutional Chip 4.0 from Perkin Elmer. Reactions were performed using SYBR Green I PCR Master Mix (Applied Biosystems), which includes the internal reference (ROX). Each qPCR reaction comprised 12.5 μl 2× SYBR Green PCR Master Mix, forward and reverse primer at optimized concentrations of 800 nM (final concentration) and 400 nM (final concentration) for the reference primers (GAPDH: TCTTCATCACCACAGAGAACTTGC and GACCTGGAAGTCACTGGGCA), and 10 ng/μl genomic. DNA template and sterile water up to a final volume of 25 μl.

The qPCR reactions were performed using the qTower (AnalytikJena, Germany). qPCR data were obtained by using the qPCRsoft software (AnalytikJena, Germany). qPCR data was normalized adapting the method described by Weksberg et al. (2005)^9^. Fold copy number (ΔKC_t_) was obtained using the formulas: ΔKC_t_ = KC_t/control_ − KC_t/affected_ and KC_ti_ = (((AC_tR_ − C_tRi_/S_R_)) × S_T_) + C_tTi_ where AC_tR_ is the average C_t_ value for the reference primer for all the samples, C_tRi_ is the C_t_ value for reference primer for the sample to be corrected, S_R_ is the slope value (from the standard curve) for the reference primer, S_T_ is the slope value for test primer, C_tTi_ is the C_t_ value for test primer.

### ELISA experiments

Microtiter plate was coated with capture antibody overnight at 4°C. After 3 washes with PBS/Tween buffer (PBS pH 7.2–7.4, Tween-20, 0.05%), microtiter plate was blocked with 200 μl/well of blocking buffer (PBS pH 7.2, 10% Fetal calf serum) for 30 min at room temperature. After 3 washes in PBS/Tween buffer, samples are incubated for overnight at 4°C. After 3 washes in PBS/Tween buffer, detection antibody is incubated at the concentration of 2 μg/ml in 100 μl blocking buffer for 1 h at room temperature. Revelation is performed by incubating 50 μl/well of alkaline phosphatase conjugated secondary antibody diluted to 1:500 in blocking buffer at room temperature for 1 h. Wells are then washed three times with PBS/Tween buffer and once with diethanolamine buffer (10 mM diethanolamine, 0.5 mM MgCl_2_ (pH 9.5) prior to pNPP substrate (Santa Cruz) addition in diethanolamine buffer to a final concentration of 1 mg/ml. Reaction is stopped by adding 0.1 M EDTA and read on microtiter plate reader at OD 405/490. Results are illustrated in graphs. In these graphs, the control condition (cells transfected with pCT) was normalized to 1 and experimental condition was (cells transfected with pUP) compared to this.

## Author Contributions

P.F.C. initiated the idea and supervised the finding of the present work. R.P., E.B., L.L. and E.H. realized the experiments from Met5A, MCF10A, Wi38 and Astro#40 cells, respectively. F.M.V., L.L. and P.F.C. discussed the results and wrote the paper.

## Supplementary Material

Supplementary Information Supplementary document
