# Supplementary material for: The DNMT1/PCNA/UHRF1 disruption induces tumorigenesis characterized by similar genetic and epigenetic signatures
Source: Sci Rep. 2014 Mar 18;4:4230. doi: 10.1038/srep04230 (PMC3957150; doi:10.1038/srep04230)
Supplement: Supplementary Information — Supplementary document [file srep04230-s1.pdf]

The Dnmt1/PCNA/UHRF1 disruption induces tumorigenesis characterized by similar genetic and epigenetic signatures.

Romain Pacaud[1-2], Emeline Brocard[1-2], Lisenn Lalier[1-2-4], Eric Hervouet[3], François M. Vallette[1-2-4] and Pierre-François Cartron[1-2-4-5*]

[1] Centre de Recherche en Cancérologie Nantes-Angers, INSERM, U892, Equipe Apoptose et progression tumorale, Equipe labellisée Ligue Nationale Contre le Cancer. 8 quai moncousu, BP7021, 44007 Nantes, France.
[2] Université de Nantes, Faculté de Médecine, Département de Recherche en Cancérologie, IFR26, F-4400, Nantes, France.
[3] Université de Franche-Comté, Equipe EA3922, 16 Route de Gray, 25035 Besançon Cedex, France.
[4] LaBCT, Institut de Cancérologie de l'Ouest, Boulevard J Monod, 44805 Nantes, Saint Herblain Cedex, France.
[5] Membre du réseau Epigénétique du Cancéropole Grand-Ouest.

[*] correspondence : pierre-francois.cartron@univ-nantes.fr, Institut de Recherche Thérapeutique INSERM U892 – CRCNA, Equipe 9 –Apoptose et Progression tumorale, 8 Quai Moncousu, BP 70721, 44007 Nantes Cedex 1 France, phone: 33-22-808-0327, fax: 33-22-808-0324

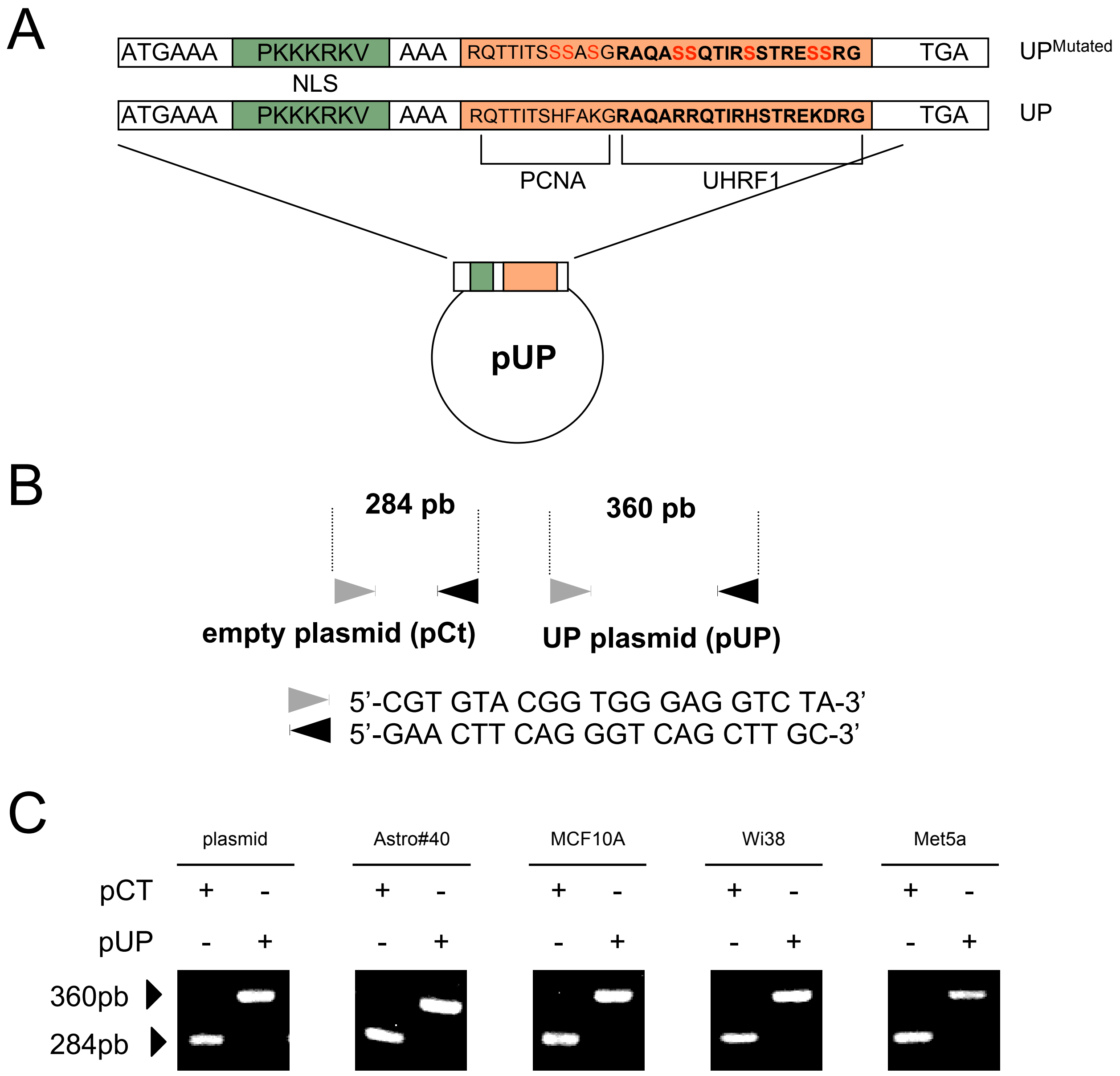

Figure S1.

Illustration of the pCt (control) and pUP transfections in cells.

A: Schematic representation of the pUP plasmid.

B: Position of primers and length of PCR products obtained after amplification of void vector or integrated UP insert.

C: Detection of the integration of insert/constructs in vector and in cells using PCR.

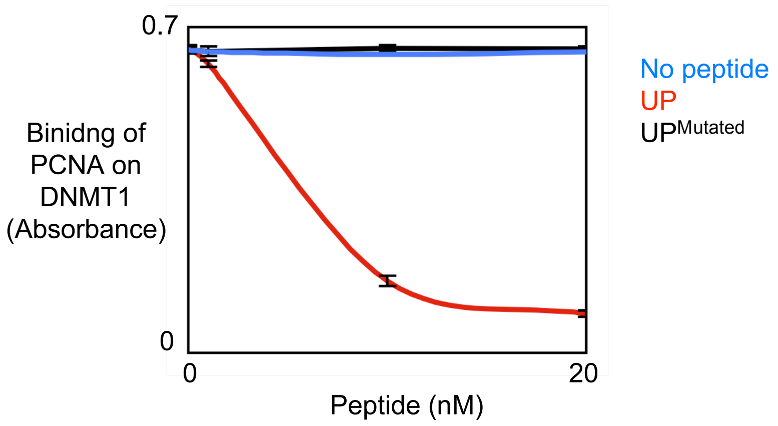

Figure S2.

Impact of the UP and UP^{mutated} peptide on the DNMT1/PCNA interaction.

Graphs illustrate the impact of peptides on specific DNMT1/DNMT1 Binding Protein (D1BP) interactions seen in Competitive Acellular Binding Assay (CABA). Graphs illustrate the average±SD of 3 independent experiments. CABA is a method permitting to measure the ability of peptides to inhibit the interaction between DNMT1 and DNMT1-binding protein. Briefly, the protein interacting (PCNA, here) with DNMT1 were incubated at a final concentration of 10 nM with varying concentrations of synthetic peptide for 2 h in running buffer (10 mM Hepes, 150 mM NaCl, 3.4 mM EDTA, 0.005% (v/v) Tween-20, pH 7.2). Next, the previous preparation was incubated with immobilized His-tagged DNMT1. The specific binding of the protein of interest to the immobilized DNMT1 in the presence and absence of peptides was quantified by subtracting the absorbance from test realized in absence of DNMT1 from that obtained in presence of DNMT1. The binding of the GST-protein of interest to the immobilized DNMT1 was determined by using a GST Antibody HRP Conjugated. iMark Microplate Absorbance Reader (BioRad, France) was used to register the absorbance.

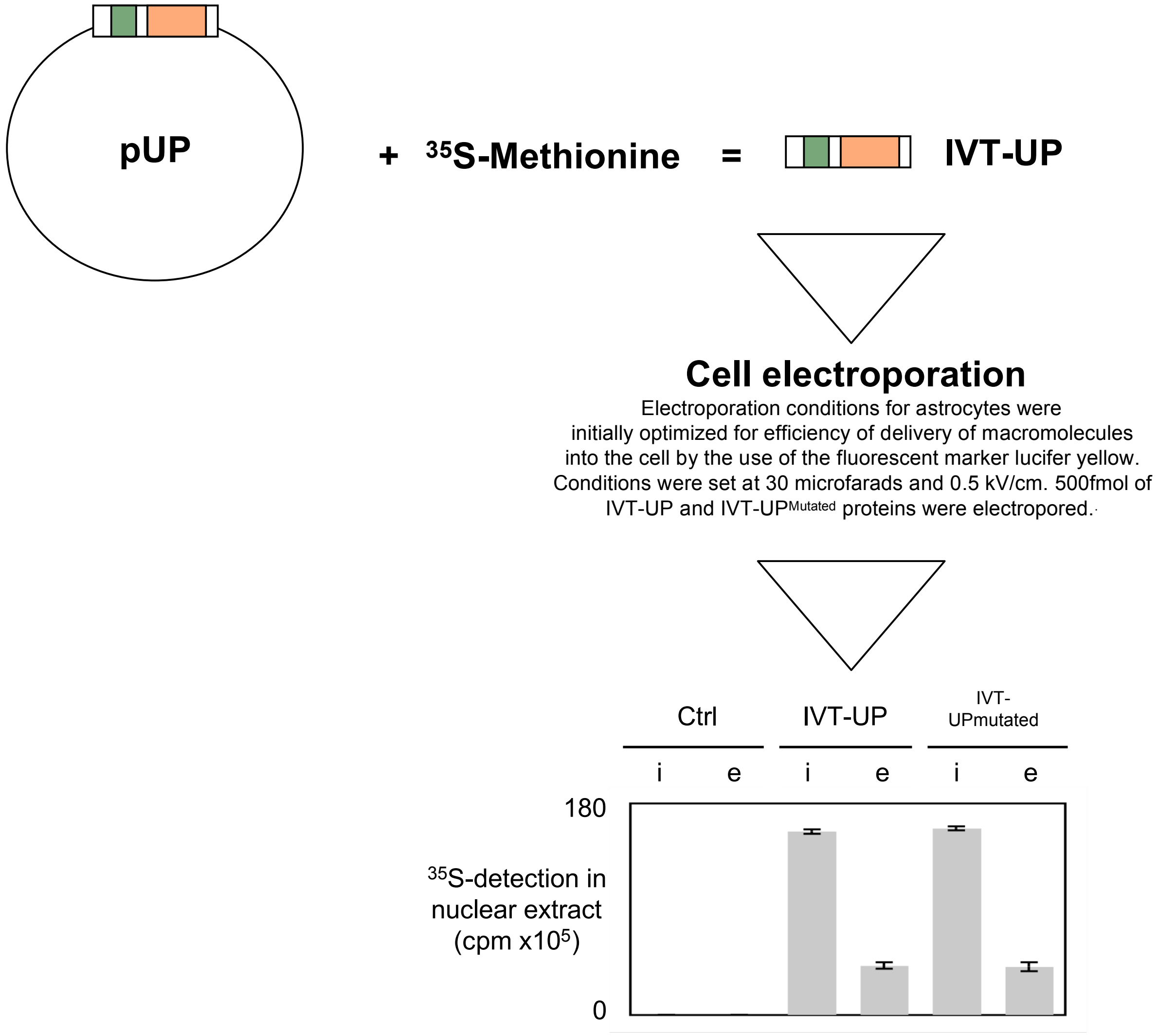

Figure S3.

Localization of UP and UP^{mutated} proteins.

The UP and UP^{mutated} proteins were obtained by using the TNT® Coupled Reticulocyte Lysate System in presence of ^{35}S-radiolabelled methionine in order to obtain radiolabelled UP and UP^{mutated} proteins (Promega, France). *In vitro* translated (IVT) UP and UP^{mutated} proteins or reticulocyte lysat were electroporated in astrocytes. 48h after electroporation, nuclear extract (Nuclear Extract Kit, Active Motif, France) was realized and analyzed to detect the presence in nucleus of radiolabelled proteins. Graphs illustrate the average±SD of 3 independent experiments. i: input and e: nuclear extract.

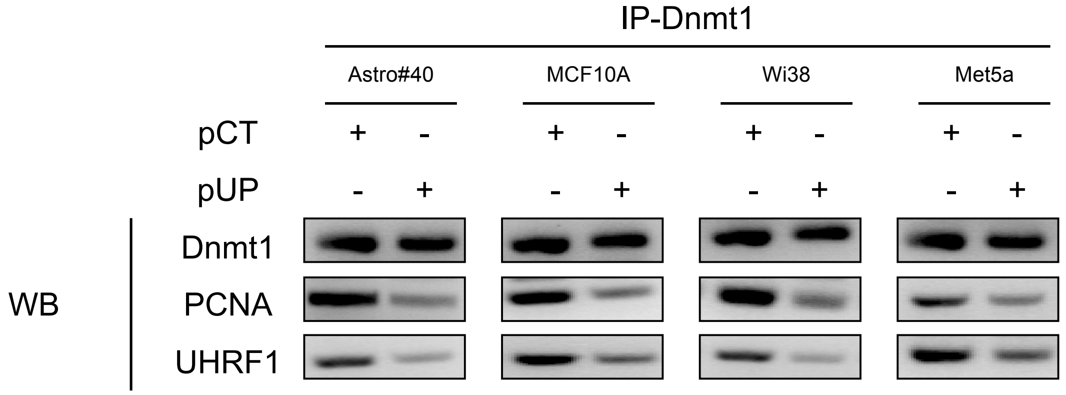

Figure S4.

Impact of the pUP transfection on the DNMT1/PCNA/UHRF1 complex.

The presence of DNMT1/PCNA/UHRF1 complex was monitored by co-immunoprecipitation experiments using the Catch and Release® v2.0 Reversible Immunoprecipitation System (Millipore, France) with 4 µg of the respective antibodies. Nuclear extracts were prepared by using the Nuclear Complex Co-IP Kit (Active Motif, France). After elution, Proteins were size fractionated by sodium dodecyl sulfate-polyacrylamide gel electrophoresis and transferred onto a nitrocellulose or PVDF membrane. Saturation and blotting were realized using the SNAP i.d™ Protein Detection System (Millipore, France). The detection of proteins was performed using ECL™(Amersham Biosciences, France). Pictures were obtained by using ChemiDoc™ Imaging system (Bio-Rad, France) and the Image Lab 4.1 software (Bio-Rad, France).

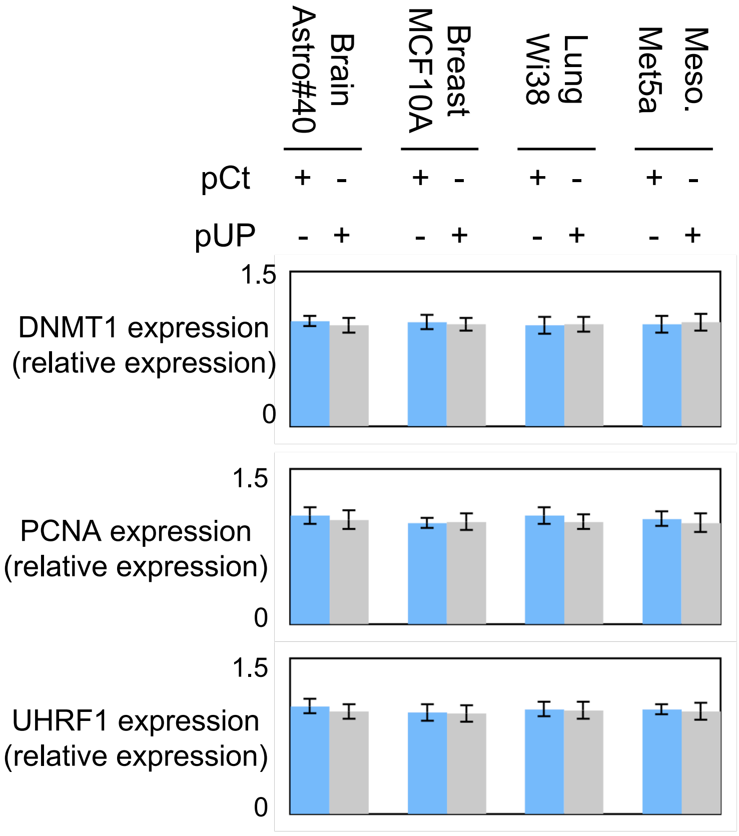

Figure S5. Impact of the expression of the pUP plasmid on the expression level of the DNMT1, UHRF1 and PCNA proteins.

ELISAs were realized to analyze the DNMT1, UHRF1 and PCNA expression.

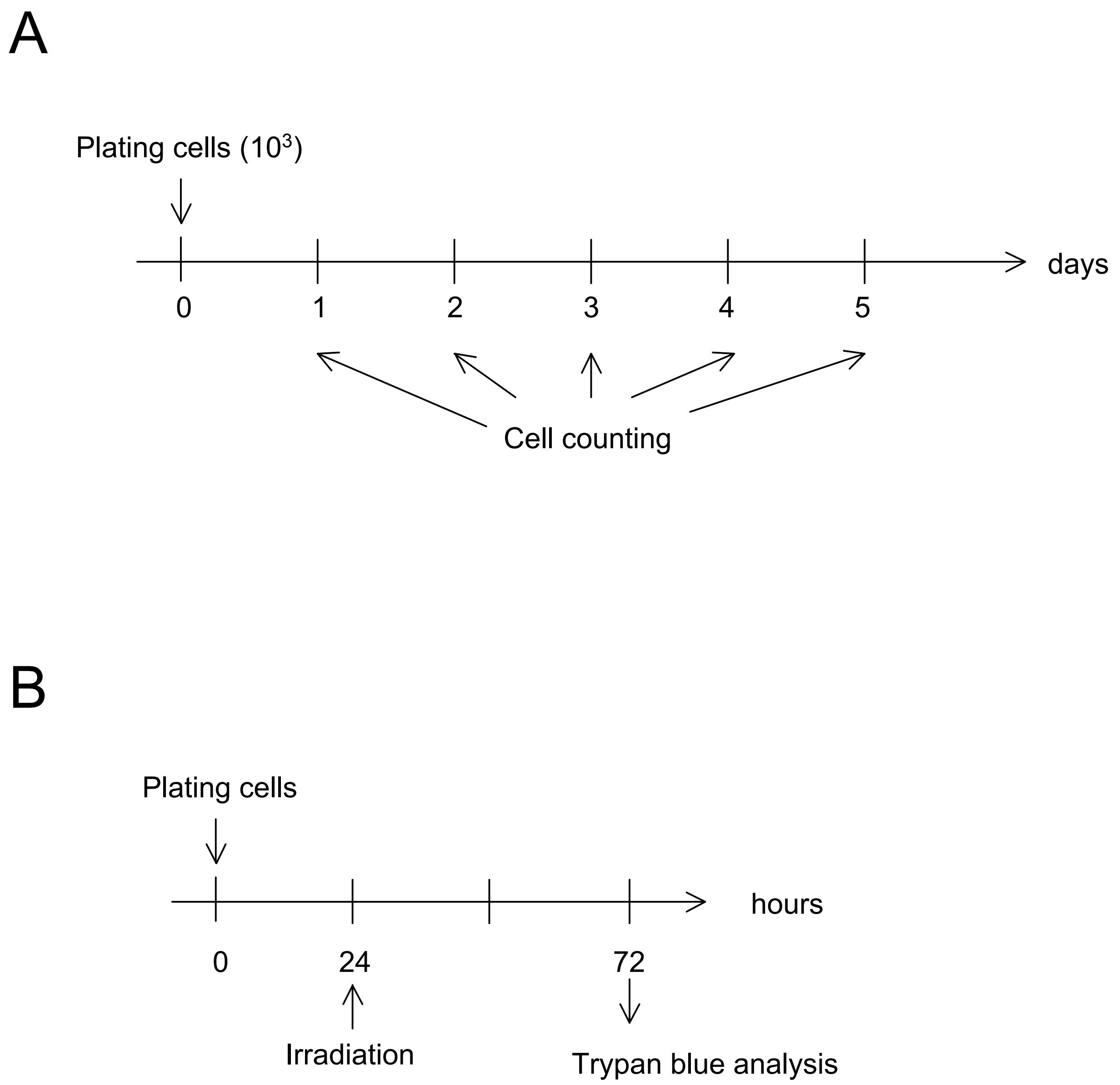

Figure S6.

Schematic representation of experiments measuring the doubling time (A)and the cell death percentage in response to irradiation treatment (B).

The cell doubling time was calculated by plating 10^3 cells at day#0 and that the cell number was counted each day during 5 days.

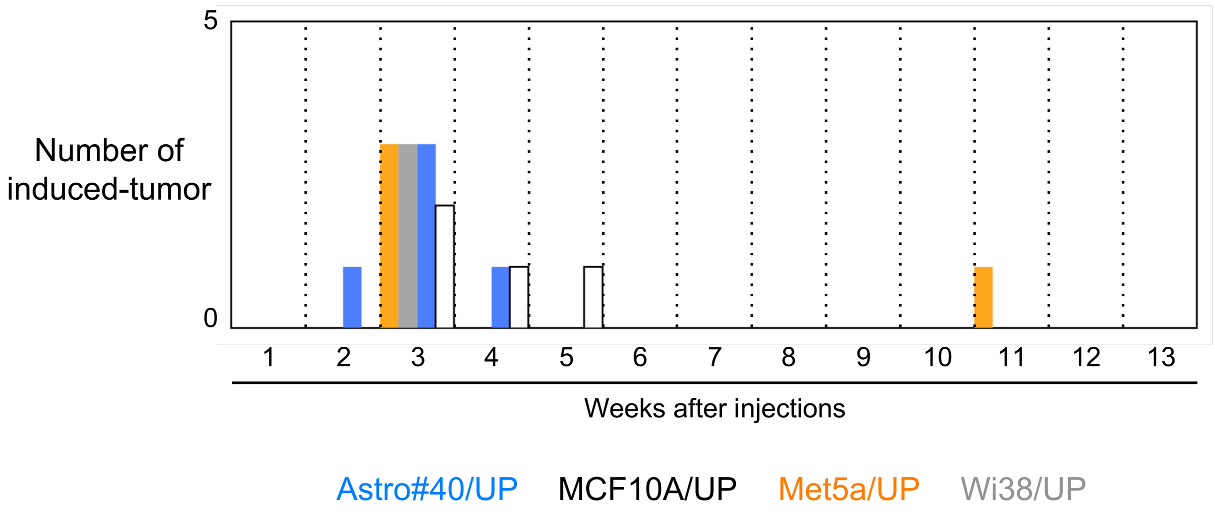

Figure S7.

Graph illustrating the kinetic of tumor formation.

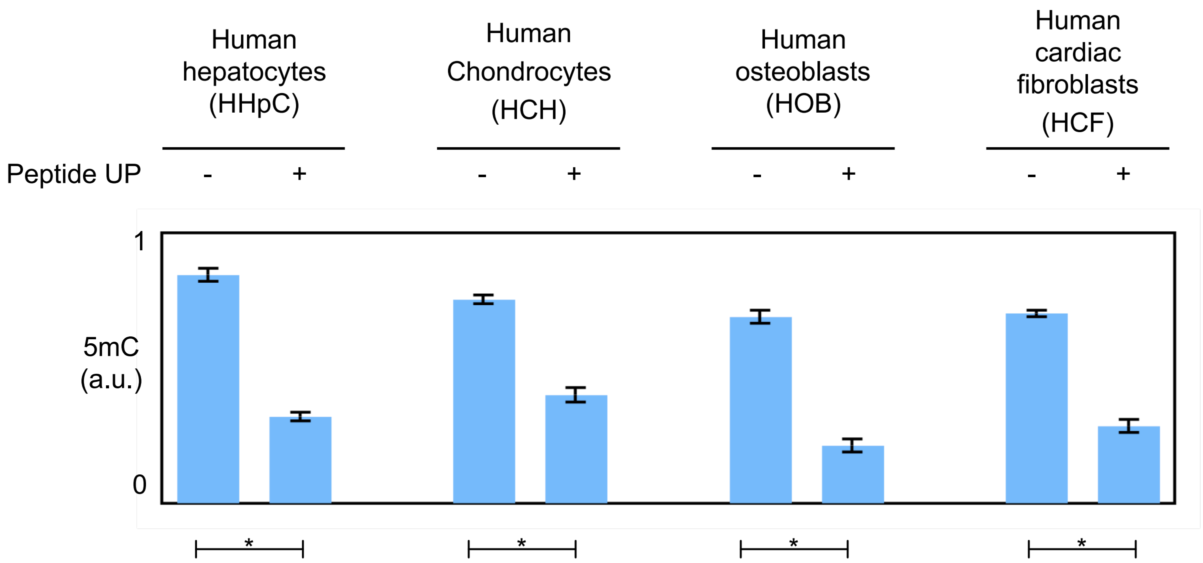

Figure S8.

Impact of the peptide-UP treatment on the level of 5-methylcytosine (5mC). Indicated cells (PromoCells, France) were treated with 1µM of peptide-UP daily during two weeks. The level of 5methylcytosine (5mC) was evaluated by ELISA method (Methylamp Global DNA Methylation Quantification kit, Epigentek-Euromedex, France). *: $p<0.05$

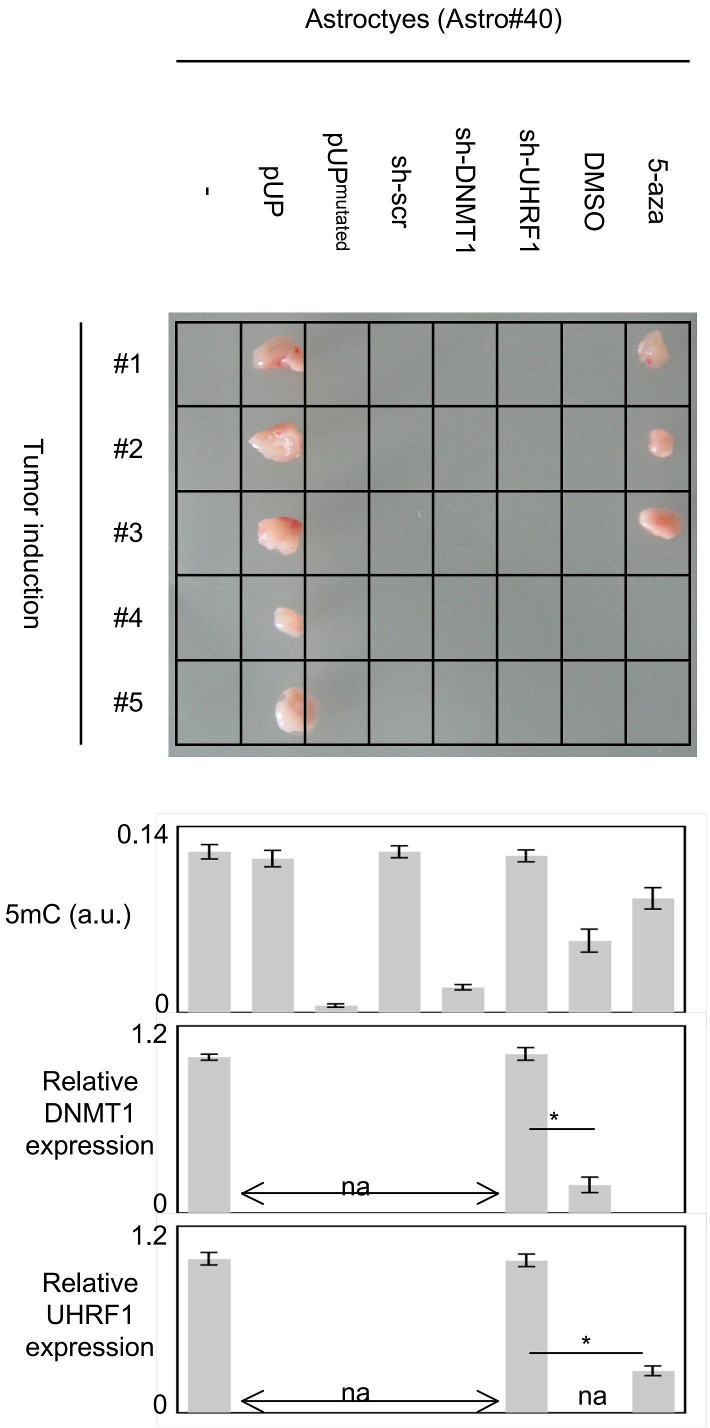

Figure S9. Impact of different strategies targeting the DNMT1 on the tumor transformation of astrocytes, and on the 5mC level.

Astrocytes transfected with pUP, pUP^{mutated}, with sh-RNA targeting DNMT1 or UHRF1 were cultivated during 4 weeks previous to be injected in subcutaneous, in Swiss nude mice (10^6 cells). Astrocytes were treated each 5 days during 4 weeks with 10μM of 5-aza previous to be injected in subcutaneous, in Swiss nude mice (10^6 cells). Picture illustrates the tumor developed or not after injections. 5 injections were performed by experimental condition.

Global level of 5-methylcytosine (5mC) was estimated by ELISA, for each experimental condition, after 4 weeks of treatment or cell culture i.e. before the cell injection in Swiss nude mice.

Expression level of DNMT1 and UHRF1 was monitored by ELISA. na: not analyzed. *: $p<0.05$

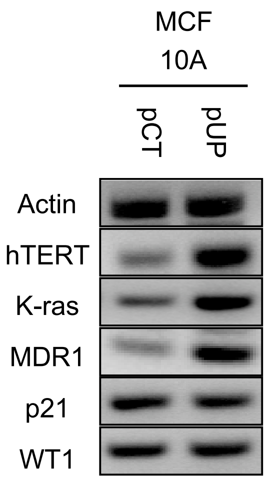

Figure S10.

Western blots analyzing the proteins expression encoded by genes seen as being hypomethylated in MCF10A-UP cells in comparison with MCF10A-Ct cells.

After extraction (RIPA buffer), proteins were size fractionated by sodium dodecyl sulfate-polyacrylamide gel electrophoresis and transferred onto a nitrocellulose or PVDF membrane. Saturation and blotting were realized using the SNAP i.d™ Protein Detection System (Millipore, France). The detection of proteins was performed using ECL™(Amersham Biosciences, France). Pictures were obtained by using ChemiDoc™ Imaging system (Bio-Rad, France) and the Image Lab 4.1 software (Bio-Rad, France).

genes	Primers		Tm (°C)
IRF7	GTTTTGGTATTTAGGTATTGGGGAT	AAACTCCCCCAACTCTTAACTCTAC	66
hTERT	TTTGGTTTTATTTTTTATTTTTTTT	CATTTCTCTTTACAAATTCTCAAAC	70
Pax6	TTGTATGTATTGTAGGGTAGAGTTGAGT	AACAAAAAAATAAAAATTAATCCTC	67
RIOK3	TTAGAATTTTTATAAAATATAGAAATAAAA	TAAAATAACTTAAAAAACAAAAAAC	61
SYBL1	GAGTAGATTTTGGTTAAGATATTTTTTGAA	TACCTAAAAACTTATTTTCTACTTTTCTA	54
WT1	TTTTTGGAGTTTATTTGTTTTTTTT	ATATAACACAACCCTCCCCC	63
KIR2DL4	TTTTTAGAAATTTTTATTTTTTTT	ATAATACAACTTCTACTACCAAAAC	61
BAGE1A	TTAGAGAAGGTTTAGAGATTTTTTTT	TATTACAAACTCCAACCTCCAACTC	62
PDGF-B	GGGTTTTTTTAGAAAATGTTGTAAAAA	AAAAAACTCCAACCTCCAAAAA	66

Supplementary table 1.

List of primers used in our experiments.

Protein	reference
Actin	Abcam#11003
hTERT	Santa Cruz sc-7215
p21	Santa Cruz sc-397
WT1	Santa Cruz sc-7385
K-ras	Santa Cruz sc-30
MDR1	Santa Cruz sc-13131
Dnmt1	Santa Cruz cs-20701 and sc-10221
PCNA	Abcam#18197 Santa cruz #sc-56
UHRF1	Santa Cruz sc-98817 and sc-100606

Supplementary table 2.

List of antibodies used in our experiments.
